# Neglected tropical disease control in a world with COVID-19: an opportunity and a necessity for innovation

**DOI:** 10.1093/trstmh/traa157

**Published:** 2020-12-24

**Authors:** Simon J Brooker, Kundai Ziumbe, Nebiyu Negussu, Siobhan Crowley, Mona Hammami

**Affiliations:** Bill & Melinda Gates Foundation, Seattle, WA, USA; Higherlife Foundation, Harare, Zimbabwe; Children's Investment Fund Foundation, Addis Ababa, Ethiopia; ELMA Philanthropies, London, UK; Crown Prince Court, Abu Dhabi, United Arab Emirates

**Keywords:** COVID-19, neglected tropical diseases, innovation

## Abstract

Countries have seen substantial disruptions to usual health services related to coronavirus disease 2019 and these are likely to have immediate and long-term indirect effects on many disease control programmes, including neglected tropical diseases (NTDs). The pandemic has highlighted the usefulness of mathematical modelling to understand the impacts of these disruptions and future control measures on progress towards 2030 NTD goals. The pandemic also provides an opportunity, and a practical necessity, to transform NTD programmes through innovation.

The coronavirus disease 2019 (COVID-19) pandemic has severely disrupted health systems and economies throughout the world and resulted in many health services and programmes being suspended, including those for neglected tropical diseases (NTDs). In April 2020, the World Health Organization (WHO) recommended that mass drug administrations (MDAs), active case-finding activities and community-based surveys for NTDs be postponed.^1^ The modelling presented by the NTD Modelling Consortium in this supplement and elsewhere^2^ provides important quantitative insights into the consequences of postponing NTD interventions and the impacts of alternative mitigation strategies that can be implemented once activities resume.

When NTD activities resume, the public health landscape will be different. NTD programmes will be operating in an altered social and economic context and they may wish to introduce novel mitigation approaches to catch up on any gains lost. To do so, programmes will need to move away from a one-size-fits-all approach and tailor intervention strategies according to local data on disease epidemiology, health systems, infrastructure and resources.

Further, NTD programmes cannot resume business as usual. There will be a need to identify innovative solutions in how interventions are planned and delivered to achieve large-scale health impact and to build programmes back better and more efficient. We argue there are four main opportunities that NTD programmes may need to evolve in a world with COVID-19: adapt, accelerate, optimize and integrate.

First and foremost, programmes will need to adapt delivery of MDAs and other interventions to communities to minimize the risk of transmission of severe acute respiratory syndrome coronavirus 2 (SARS-CoV-2). The WHO has recently provided guidance on the risk–benefit assessment process to guide decisions around resuming interventions and mitigation measures that should be applied to decrease risks.^3^ Many of these measures, such as providing community drug distributors (CDDs) with masks and gloves, screening for COVID-19 symptoms and adding crowd controls to manage social distancing, will increase operational costs. Where a fixed-post approach was previously used to distribute drugs, distribution needs to move to an adapted door-to-door approach and this in turn may encourage a shift from wide-area MDA to more data-driven, targeted approaches. We are already seeing national programmes and their implementing partners come up with innovative adaptations to enable safe delivery of interventions in a world with COVID-19, and such local solutions should be built upon.

Second, the modelling presented in this supplement highlights opportunities to accelerate progress towards the 2030 targets by intensifying the frequency or scale of WHO-recommended strategies. The impact of disrupting NTD programmes is greatest for trachoma, schistosomiasis and visceral leishmaniasis (VL) in high-transmission areas where the risk of resurgence is greatest, and the implementation of more intensive intervention strategies, such as moving to biannual or quarterly MDA for trachoma or to community-wide MDA for schistosomiasis, or active case detection activities for VL can help mitigate against missed services. Such strategies may, in defined settings, accelerate progress towards 2030 disease targets.

Even before the pandemic there was a need to accelerate progress in certain areas where infection levels remained persistently high despite multiple years of intervention; for example, persistently high levels of trachoma after 5–10 y of intervention in areas of Ethiopia.^4^ In such settings, the current modelling suggests biannual or quarterly MDA could help catch up on missed MDA rounds and help accelerate progress towards the 2030 trachoma goals.^5^ The benefits of more frequent MDA have been demonstrated with a randomized trial that found quarterly MDA targeted at children was more effective in reducing *Chlamydia trachomatis* infection compared with community-wide annual MDA.^6^ While benefits of more frequent MDA need to be balanced against cost and logistical considerations, shorter programmes are likely to yield cost savings in the long-term, as well as reduce the total amount of antibiotics required and the likelihood of antimicrobial resistance emerging.

For those diseases that have a low rate of rebound, such as lymphatic filariasis (LF), onchocerciasis and hookworm, the impact of missed MDAs is minimal and a single round of biannual or community-wide MDA will adequately compensate for a missed round of MDA. The benefits of such strategies are well established^7,8^ and offer the opportunity to move beyond current 2030 targets; for example, moving from morbidity control to interruption of transmission for soil-transmitted helminths (STHs)^9^ or schistosomiasis.^10^ However, any effort to increase the frequency and scale MDA should not replace efforts to increase coverage and reduce systematic non-compliance or improve access to water, sanitation and hygiene.

Third, programmes can improve and optimize the way they plan and deliver interventions. One example is the use of digital tools and data layers to improve microplanning for MDA, overcoming the difficulties of using paper treatment registers and maps and outdated population denominator data. Many of these tools have been effectively used by other campaigns, such as polio and routine immunization, where digital microplanning has improved the coverage, efficiency and health impact of campaigns,^11^ and these tools can readily be modified for use in NTD programmes. There are also important opportunities to apply digital solutions to the collection, analysis and use of NTD data. For example, the Geshiyaro Project in Ethiopia is using biometric fingerprint technology to identify and track participants in a project evaluating the feasibility of interrupting the transmission of STHs and schistosomiasis.^12^ Other examples include the use of ESPEN Collect to conduct subdistrict mapping to better target schistosomiasis, the TT Tracker to capture surgery data, the use of an electronic register for enumerating populations and monitoring treatment coverage^13^ and geolocation data and flow modelling tools to quantify and track population movements.

Fourth, there may be opportunities to integrate across NTDs, such as combining onchocerciasis and LF with schistosomiasis and with other health programmes. There is also a potential role for CDDs in COVID-19 contact tracing activities in communities. Integrated delivery will save time and financial and human resources in ramping up interventions and minimize the number of contacts with households. As community trust in health systems is lessened by the pandemic, stronger integration of different health campaigns could help improve community engagement and social mobilization and strengthen health systems. Such integration may leverage new funding sources, though the anticipated benefits of integrated delivery need to be balanced against an increase in programme complexity and cost.

As programmes restart population-based NTD surveys, there is an unrealized role for novel, integrated survey designs that incorporate geostatistical^14^ and adaptive sampling^15^ approaches, which can reduce the number of community visits and survey costs. The high cost of current population-based surveys is prohibitive for some programmes and will likely be more prohibitive in a resource-constrained COVID-19 environment.

In just 6 months the novel SARS-CoV2 coronavirus has affected almost everyone, infecting millions and killing hundreds of thousands, shutting down economies and disrupting essential health services and disease control programmes. The likelihood is that we will be living with this coronavirus for the foreseeable future. If there were ever a time to introduce innovative approaches into how NTD programmes are planned and implemented, surely that time is now. Our previous aspirations around innovation in NTDs have now become a practical necessity.

## Data Availability

Not applicable.

## References

[1] World Health Organization. COVID-19: WHO issues interim guidance for implementation of NTD programmes. Geneva: World Health Organization; 2020.

[2] NTD Modelling Consortium. The potential impact of programmes interruptions due to COVID-19 on 7 neglected tropical diseases: a modelling-based analysis. Gates Open Res. 2020;4:115.

[3] World Health Organization. Maintaining essential health services: operational guidance for the COVID-19 context. Geneva: World Health Organization; 2020

[4] Nash SD , Chernet A, Moncada J et al. Ocular *Chlamydia trachomatis* infection and infectious load among pre-school aged children within trachoma hyperendemic districts receiving the SAFE strategy, Amhara region, Ethiopia. PLoS Negl Trop Dis. 2020;14(5):e0008226. 32421719 10.1371/journal.pntd.0008226 PMC7259799

[5] Blumberg S , Borlase A, Prada JM et al. Implications of the COVID-19 pandemic on eliminating trachoma as a public health problem. medRxiv. 2020; doi: 10.1101/2020.10.26.20219691. 10.1093/trstmh/traa170 PMC7928550 33449114

[6] Lietman TM , Ayele B, Gebre T et al. Frequency of mass azithromycin distribution for ocular chlamydia in a trachoma endemic region of Ethiopia: a cluster randomized trial. Am J Ophthalmol. 2020;214:143–50. 32171768 10.1016/j.ajo.2020.02.019 PMC9982657

[7] Pullan RL , Halliday KE, Oswald WE et al. Effects, equity, and cost of school-based and community-wide treatment strategies for soil-transmitted helminths in Kenya: a cluster-randomised controlled trial. Lancet. 2019;393:2039–50. 31006575 10.1016/S0140-6736(18)32591-1 PMC6525786

[8] Batsa Debrah L , Klarmann-Schulz U, Osei-Mensah J et al. Comparison of repeated doses of Ivermectin versus Ivermectin Plus Albendazole for the treatment of Onchocerciasis: a randomized, open-label, clinical trial. Clin Infect Dis. 2020;71:933–43. 31536624 10.1093/cid/ciz889 PMC7428389

[9] Malizia V , Giardina F, Vegvari C et al. Modelling the impact of COVID-19-related control programme interruptions on progress towards the WHO 2030 target for soil-transmitted helminths. Trans R Soc Trop Med Hyg. 2020; doi: 10.1093/trstmh/traa156. 10.1093/trstmh/traa156 PMC7798673 33313897

[10] Kura K , Hardwick RJ, Truscott JE et al. The impact of mass drug administration on schistosoma haematobium infection: what is required to achieve morbidity control and elimination? Parasit Vectors. 2020;13:554. 33203467 10.1186/s13071-020-04409-3 PMC7672840

[11] Ali D , Levin A, Abdulkarim M et al. A cost-effectiveness analysis of traditional and geographic information system-supported microplanning approaches for routine immunization program management in northern Nigeria. Vaccine. 2020;38(6):1408–15. 31924428 10.1016/j.vaccine.2019.12.002

[12] Mekete K , Ower A, Dunn J et al. The Geshiyaro Project: a study protocol for developing a scalable model of interventions for moving towards the interruption of the transmission of soil-transmitted helminths and schistosome infections in the Wolaita zone of Ethiopia. Parasite Vectors. 2019;12(1):503. 10.1186/s13071-019-3757-4 PMC6820996 31665080

[13] Oswald WE , Kennedy DS, Farzana J et al. Development and application of an electronic treatment register: a system for enumerating populations and monitoring treatment during mass drug administration. Glob Health Action. 2020;13(1):1785146. 32666905 10.1080/16549716.2020.1785146 PMC7480461

[14] Fronterre C , Amoah B, Giorgi E et al. Design and analysis of elimination surveys for neglected tropical diseases. J Infect Dis. 2020;221(Suppl 5):S554–60. 31930383 10.1093/infdis/jiz554 PMC7289555

[15] Andrade-Pacheco R , Rerolle F, Lemoine J et al. Finding hotspots: development of an adaptive spatial sampling approach. Sci Rep. 2020;10(1):10939. 32616757 10.1038/s41598-020-67666-3 PMC7331748

